# Biosynthesis of Antimicrobial Ornithine-Containing Lacticin 481 Analogues by Use of a Combinatorial Biosynthetic Pathway in *Escherichia coli*

**DOI:** 10.1021/acssynbio.4c00650

**Published:** 2024-12-11

**Authors:** Yanli Xu, Roos Reuvekamp, Oscar P. Kuipers

**Affiliations:** Department of Molecular Genetics, Groningen Biomolecular Sciences and Biotechnology Institute, University of Groningen, Groningen 9747 AG, The Netherlands

**Keywords:** lacticin 481, lanthipeptide bioengineering, SyncM, ornithine, OspR, leader peptide

## Abstract

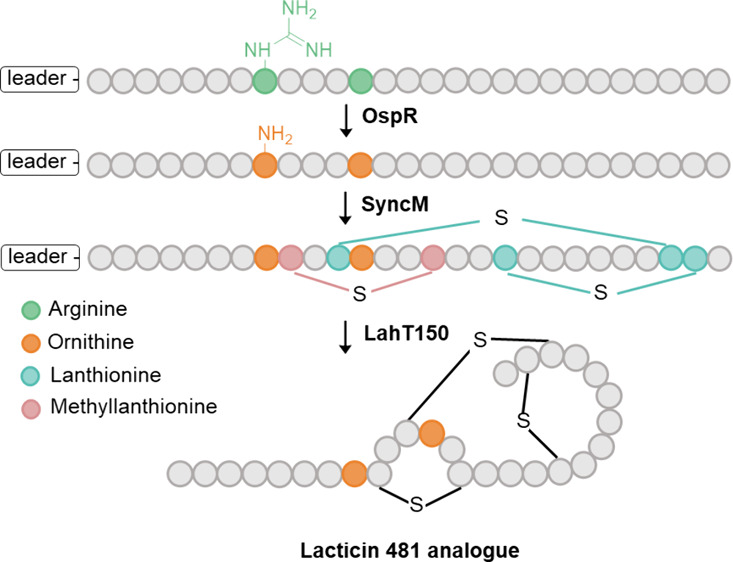

Lacticin 481, a ribosomally synthesized and post-translationally modified peptide (RiPP), exhibits antimicrobial activity, for which its characteristic lanthionine and methyllanthionine ring structures are essential. The post-translational introduction of (methyl)lanthionines in lacticin 481 is catalyzed by the enzyme LctM. In addition to macrocycle formation, various other post-translational modifications can enhance and modulate the chemical and functional diversity of antimicrobial peptides. The incorporation of noncanonical amino acids, occurring in many nonribosomal peptides (NRPs), is a valuable strategy to improve the properties of antimicrobial peptides. Ornithine, a noncanonical amino acid, can be integrated into RiPPs through the conversion of arginine residues by the newly characterized peptide arginase OspR. Recently, a flexible expression system was described for engineering lanthipeptides using the post-translational modification enzyme SyncM, which has a relaxed substrate specificity. This study demonstrates that SyncM is able to catalyze the production of active lacticin 481 by recognition of a designed hybrid leader peptide, which enables the incorporation of both ornithine and (methyl)lanthionine. Utilizing this hybrid leader peptide, the functional order was established for the production of active ornithine-containing lacticin 481 analogues at positions 8 and 12 *in vivo*. Furthermore, this study demonstrates that prior lanthionine (Lan) and methyllanthionine (MeLan) formation may preclude ornithine incorporation at specific sites of lacticin 481. The antibacterial activity of ornithine-containing lacticin 481 analogues was evaluated using *Bacillus subtilis* as the indicator strain. Overall, the synthetic biology pathway constructed here helped to elucidate aspects of the substrate preferences of OspR and SyncM, offering practical guidance to combine these modifications for further lantibiotic bioengineering.

## Introduction

**Figure 1:**
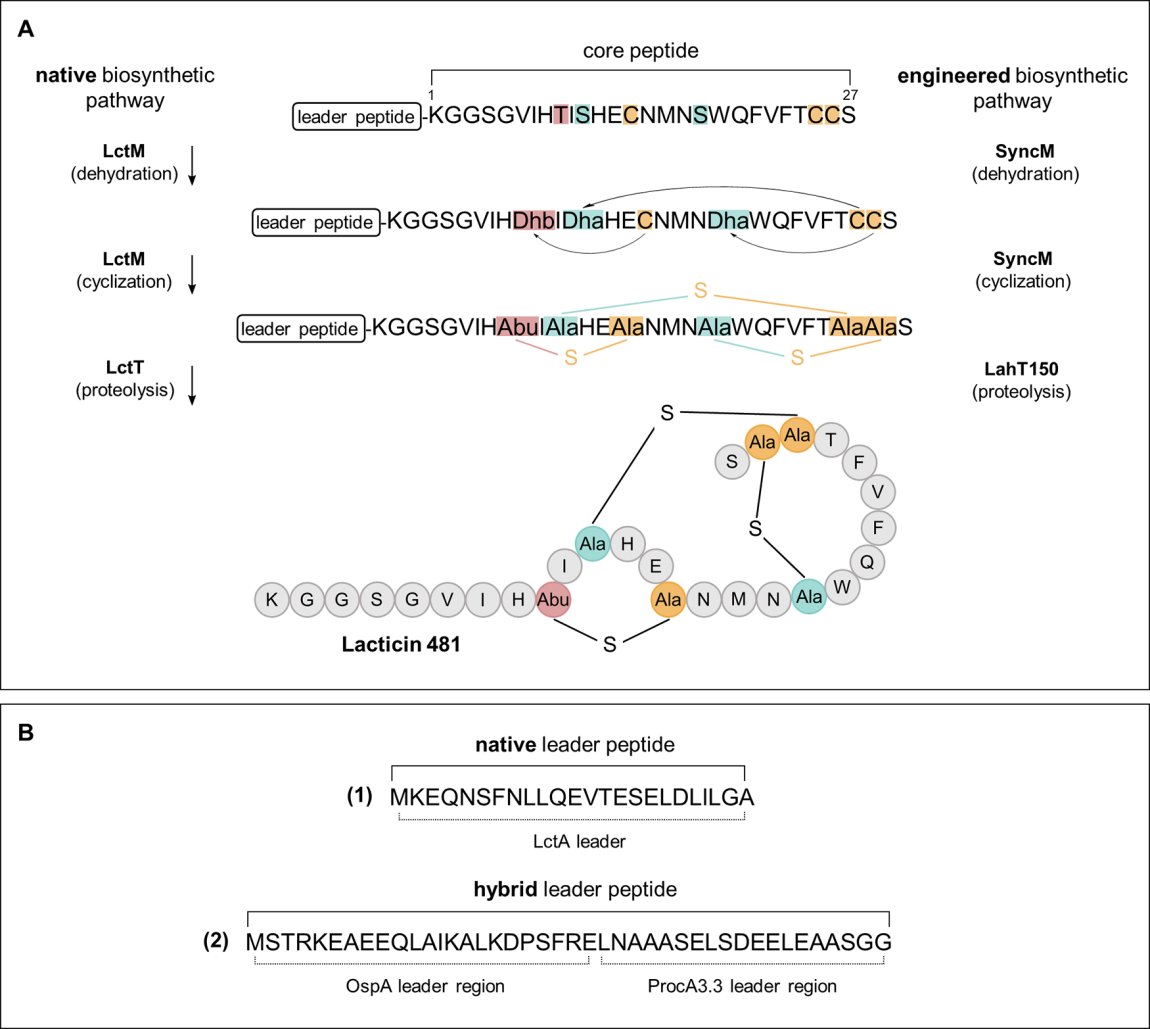
Biosynthesis of lacticin 481.^26^**A)** The biosynthetic pathway of lacticin 481, showing the difference between the enzymes used in the native pathway and the engineered biosynthetic pathway presented in this study. SyncM (instead of LctM) catalyzes the dehydration of Ser/Thr to Dha/Dhb and the subsequent cyclization with Cys thiol groups in the core peptide of the precursor. Proteolytic cleavage of the leader peptide by LahT150 (instead of LctT) results in the mature, active peptide. Only Dhb/Dha involved in correct ring formation are highlighted. **B)** Sequences of the native LctA leader peptide and the hybrid leader peptide designed in this study for recognition by OspR and SyncM.

**Figure 2:**
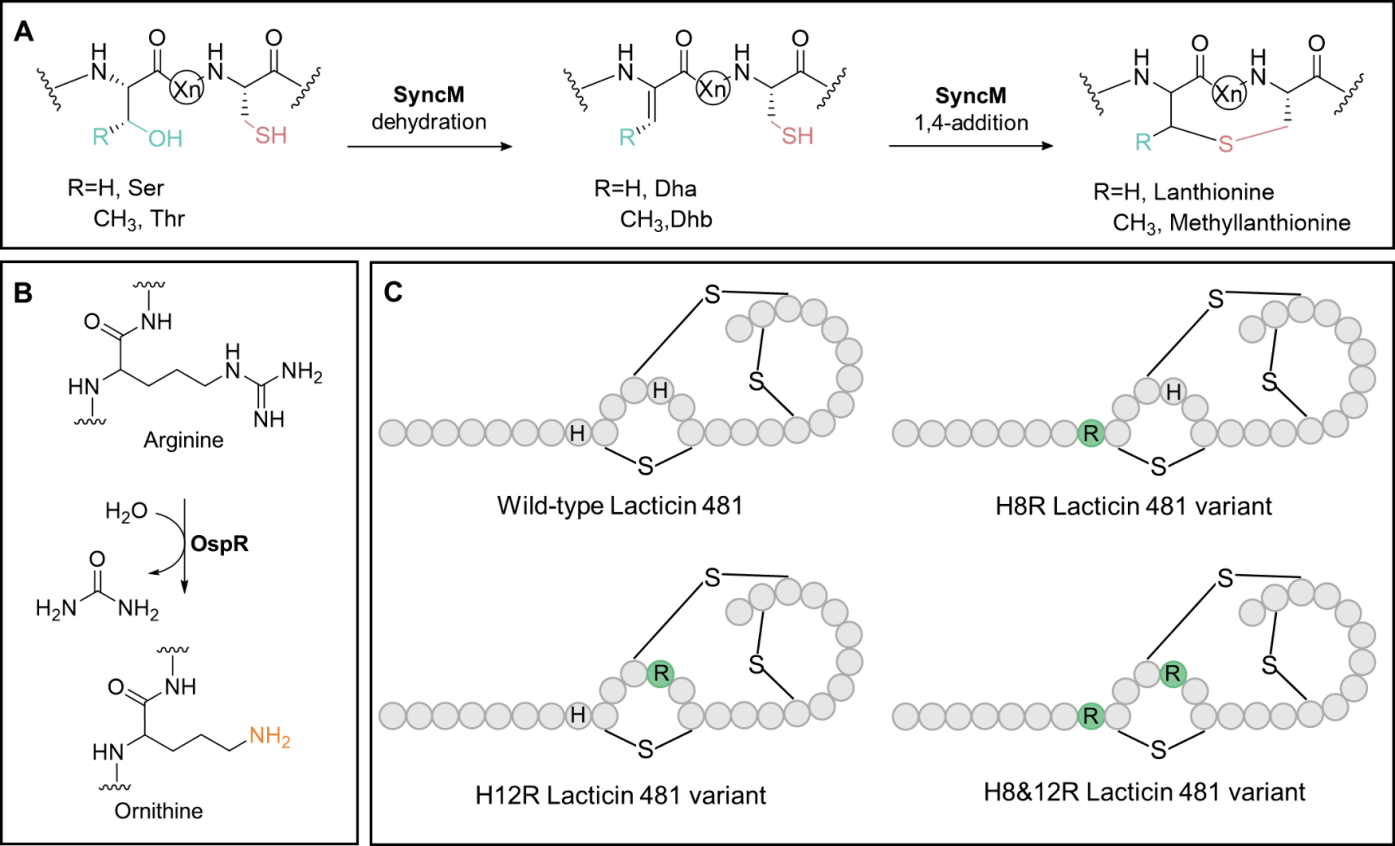
Modifications in lacticin 481. **A)** Initial dehydration of Ser/Thr residues to dehydroalanine (Dha) and dehydrobutyrine (Dhb), followed by stereoselective addition of Cys thiol groups to these residues resulting in the formation of lanthionine (Lan) and methyllanthionine (MeLan) rings.^18^**B)** The modification of arginine to ornithine and urea by OspR.^43^**C)** The lacticin 481 analogues produced in this study compared to the wild-type peptide. To study the effect of ornithine residues on (Me)Lan ring formation, histidine residues on position 8 and/or 12 are replaced by arginine residues, which can be modified by OspR.

Lanthipeptides are ribosomally encoded and post-translationally modified peptides (RiPPs) with antifungal, antiviral and immunomodulatory activities.^1−5^ Many lanthipeptides exhibit antimicrobial activity (and are then called lantibiotics) against pathogenic bacteria, including strains that have developed resistance to antibiotics.^6−11^ The family of lanthipeptides is classified into five classes based on their modification enzymes.^12,13^ Lacticin 481 is a class II lantibiotic and is derived from a gene-encoded precursor peptide, which consists of an N-terminal leader peptide and a C-terminal modifiable core peptide (Figure 1A).^14,15^ Lanthipeptides are characterized by the presence of lanthionine (Lan) and/or methyllanthionine (MeLan) ring structures. These are formed in a two-step process (Figure 1A, Figure 2A). First, dehydration of serine (S) and threonine (T) residues results in dehydroalanine (Dha) and dehydrobutyrine (Dhb). The intramolecular thioether cross-links are then formed by stereoselective 1,4-addition of cysteine thiol groups to the dehydroamino acids. (Me)Lan residues contribute to the stability of the bioactive conformation of lantibiotics and can provide protection against proteases.^16^ After these modifications, the peptide is exported, followed by cleavage of the leader by a protease, resulting in the mature, active peptide (Figure 1A).^4^ For class II lantibiotics, like lacticin 481, the dehydration and cyclization steps in the native biosynthetic pathway are performed by a single bifunctional LctM enzyme.^17^ Many lantibiotic biosynthetic enzymes demonstrate a considerable tolerance toward sequence variations in their peptide substrates.^18^ However, some (methyl)lanthionine-introducing enzymes exhibit a moderate to high substrate specificity, which can restrict their capacity to modify core peptides that are unrelated to their native substrate(s).^19^ A new group of lanthipeptide synthetases called ProcM-like enzymes show high substrate promiscuity.^20,21^ Modification of a hybrid precursor by ProcM yields bioactive lacticin 481.^22^ Recently, SyncM was identified as the most promiscuous lanthipeptide synthetase described to date,^23^ and the flexibility of a corresponding expression system was demonstrated.^19^ SyncM’s relaxed substrate specificity toward precursor peptides implies its potential for the production of (novel) lanthipeptides with additional modifications and modulated and/or improved properties. Though lantibiotics hold promise as therapeutic agents with excellent pharmacodynamics, particularly against clinically resistant bacterial strains, their pharmacokinetics may be suboptimal.^24,25^

Several efforts have been made to achieve a larger chemical diversity in lantibiotics. These efforts aimed to enhance their biological and physicochemical properties, in particular through the introduction of additional post-translational modifications.^14,27^ The incorporation of noncanonical amino acids (or nonproteinogenic or unnatural amino acids) is a valuable approach in this respect and can even result in the introduction of novel bioactivities.^27−29^ Several studies have shown the potential of incorporating noncanonical amino acids in lanthipeptides, for example *in vivo* using genetic code expansion strategies,^28−33^ or *in vitro* using solid-phase peptide synthesis (SPSS).^34,35^ Previous work by Levengood et al. demonstrated that introducing noncanonical amino acids in core peptides prepared *in vitro* by a mutasynthetic approach improved the bioactivity of lacticin 481.^36^ The introduction of noncanonical amino acids was also found to increase the peptide’s affinity to its target lipid II, thereby inhibiting the transglycosylation reaction essential for cell wall formation.^37^

Ornithine is a noncanonical amino acid that is commonly found in nonribosomal peptides (NRPs), such as daptomycin, gramicidin S and tyrocidine.^38−40^ Until recently, the presence of ornithine was thought to be limited to NRPs. However, recent research has discovered ornithine residues in the RiPP natural product landornamide A.^41^ The peptide arginase OspR post-translationally hydrolyzes arginine residues, thus yielding ornithine (Figure 2B). Since ornithine is derived from the canonical amino acid arginine, ornithine can be incorporated into peptides *in vivo* without the expression of an additional biosynthetic translation machinery. OspR is considered highly promiscuous, which constitutes opportunities for peptide bioengineering.^42,43^ Ornithine can undergo further enzymatic or chemical modifications, with additional bioengineering benefits for peptides.^44,45^ Studies have demonstrated that the incorporation of ornithine enhances proteolytic resistance to a dodecapeptide.^46^ The incorporated ornithines could serve as a starting point for further modifications. More importantly, this study presents a methodology for the incorporation of noncanonical amino acids, such as ornithine, in a lanthipeptide, enabling future advancements in peptide engineering. The incorporation of ornithines in lanthipeptides, may, depending on the exact site of incorporation in particular in noncyclized parts remote from cyclized structures of the peptide, contribute to enhanced stability. For instance, these sites will be less prone to proteolytic degradation, e.g. by trypsin. Considering the fact that Orn residues are frequently observed in strong antimicrobial NRPs, where they are known in specific cases to be important for activity, we can also speculate that Orn at specific positions might improve antimicrobial activity. Future research will be conducted to test this hypothesis.

**Figure 3:**
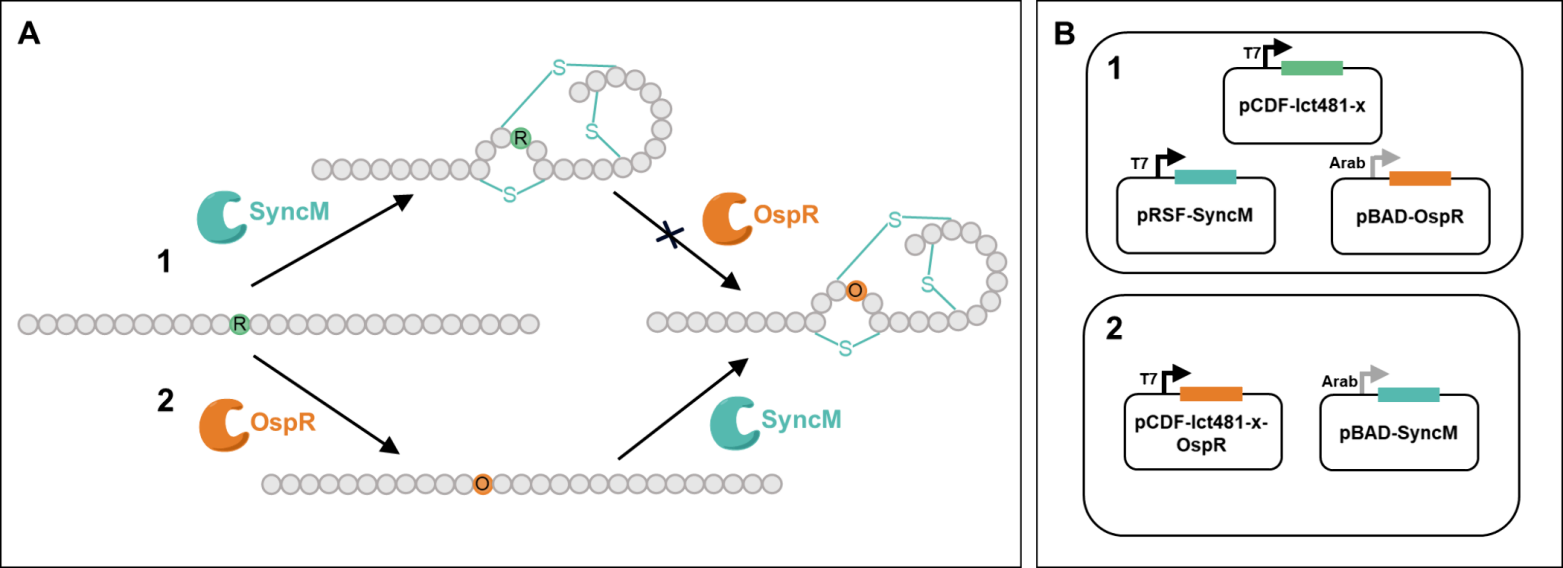
Expression systems used in this study. **A)** Proposed routes of modification for lacticin 481, based on the order of expression of the modification enzymes SyncM and OspR. Only expression of the precursor peptide with OspR first, followed by expression of SyncM (pathway 2) led to ornithine-containing lacticin 481 analogues. **B)** Schematic overview of the plasmids used for the two different expression systems. The x represents either wt (wild-type), H8R, H12R or H8&12R lacticin 481 (lct481) analogue.

In this study, we present an engineered combinatorial biosynthetic pathway (Figure 1A) for the production of bioactive ornithine-containing lacticin 481 analogues in *Escherichia coli*. The hybrid leader sequence (Figure 1B (2)), initially designed for recognition by the SyncM and OspD enzymes,^47^ was repurposed in this study to enable recognition by both SyncM and OspR. To investigate whether incorporating ornithine residues affects the formation of (Me)Lan rings with the correct stereochemical configuration, leading to bioactive peptides, here, position 8 and position 12 were chosen because position 8 is adjacent to the MeLan ring while position 12 is within the Melan ring and adjacent to the Lan ring. These two positions will provide a basis to assess the impact of this noncanonical amino acid on (methyl)lanthionine ring formation. The study thus aims at establishing the generality of the method. For this purpose, we introduced arginine residues on position 8 and 12 replacing the histidine amino acids with basic arginines (Figure 2C). Two expression systems were used to explore the preferred order of combinatorial post-translational modifications by SyncM and OspR (Figure 3A/B). We demonstrate SyncM’s substrate tolerance toward ornithine-containing lacticin 481 analogues, and prove that OspR prefers linear lantibiotic core peptide substrates. The feasible order of complete modification is first modification by OspR and thereafter modification by SyncM. This study sheds light on the combinatorial application of SyncM and OspR with a hybrid leader peptide as an additional tool for engineering lantibiotics, enabling further chemical diversification of antimicrobial peptides.

## Results and Discussion

### (Methyl)lanthionine Formation in Wild-Type Lacticin 481 by SyncM with a Hybrid Leader

**Figure 4:**
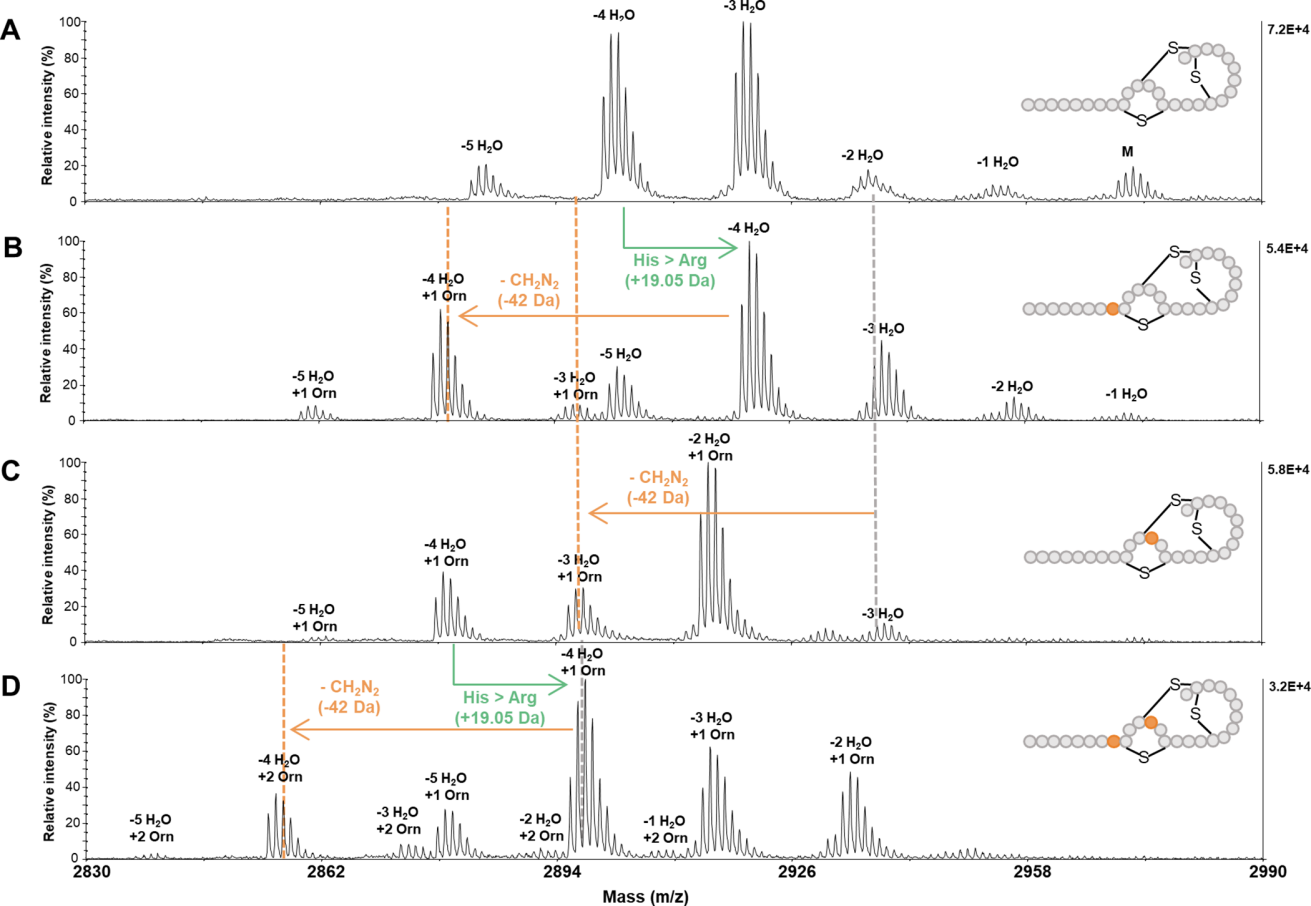
MALDI-TOF mass analysis of lacticin 481 analogues produced by expression of OspR first, followed by SyncM. The spectra are from a HPLC purified fraction of each analogue. Modification of the lacticin 481 analogues by SyncM and OspR is observed. MALDI-TOF mass spectra of **A)** wild-type lacticin 481, **B)** the H8R lacticin 481 analogue showing the shift caused by the substitution of histidine to arginine, and modification by OspR, **C)** the H12R lacticin 481 analogue depicting modification by OspR, and **D)** the H8&12R lacticin 481 analogue showing the substitution of a second histidine to arginine, and modification by OspR.

**Figure 5:**
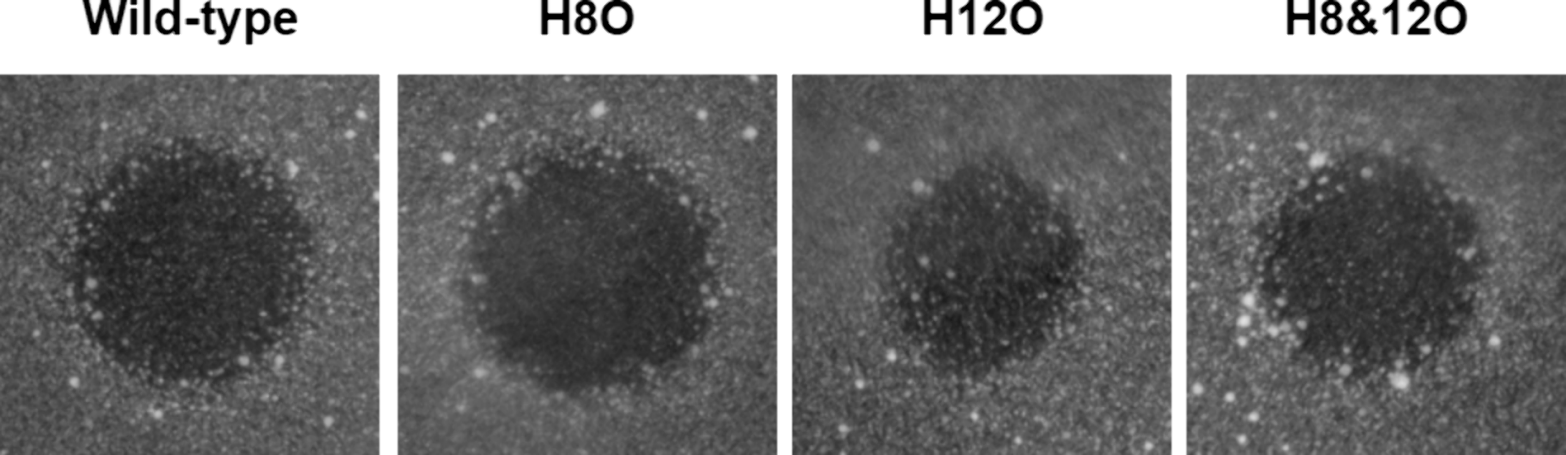
Antimicrobial activity test of the ornithine-containing lacticin 481 analogues against *Bacillus subtilis 168*. The full plate including positive and negative controls is available in the Supporting Information (Figure S10).

The leader recognition sequence for SyncM has not yet been established, but the SyncM homologue ProcM, with a ProcA3.2 leader peptide fused to the lacticin 481 core peptide, can produce active lacticin 481.^22^ Here, we used the recognition sequence of ProcM’s native substrate, ProcA3.3, for the generation of the hybrid leader (Figure 1B (2)). Specifically, we used the C-terminal leader region of the ProcA3.3 leader peptide, known to be important for recognition by ProcM,^4,48^ to evaluate SyncM’s modification ability. We first heterologously coexpressed the gene encoding the wild-type lacticin 481 precursor peptide attached to this hybrid leader and the lanthipeptide synthetase SyncM in *E. coli*. Analysis by MALDI-TOF mass spectrometry displayed peptide products with expected masses corresponding to various dehydration states (Figure 4A). Subsequently, the antimicrobial activity of wild-type lacticin 481 was assessed. Previous work by van der Donk et al. demonstrated that the antimicrobial activity of lacticin 481 was completely abolished upon substitution of any of the three DL-Lan/MeLan ring structures with the corresponding LL stereoisomers. Our results show that wild-type lacticin 481 exhibits antimicrobial activity against *B. subtilis* (Figure 5). This confirms successful formation of (methyl)lanthionine rings with correct stereochemical configuration, corresponding to the active peptide product, characterized by masses representing at least three dehydrated residues. The results indicate that SyncM can indeed recognize the ProcA3.3 hybrid leader sequence for modification of the lacticin 481 core peptide with three correct (Me)Lan rings. This not only illustrates the recognition sequence of SyncM for peptide lacticin481, but also underscores SyncM’s high substrate tolerance.

### (Methyl)lanthionine Formation in Lacticin 481 Analogues by SyncM

To introduce ornithine into lacticin 481 by the conversion of arginine to ornithine by OspR, first some arginine-containing lacticin 481 analogues were generated. Positions 8 and 12 were selected, to study the effect of ornithine on stereospecific (methyl)lanthionine formation. Additionally, an analogue with mutations on both positions was generated. After successful site-directed mutagenesis and confirmation of the constructs by sequencing, we expressed these analogues (called the H8R, H12R and the H8&12R lacticin 481 analogues, respectively) and wild-type lacticin 481 in *E. coli*. The MALDI-TOF mass spectrometry results (Supplementary Figure S2 and Table S2) demonstrate that the arginine-containing lacticin 481 analogues are successfully modified by SyncM. The analogues also display antimicrobial activity against *Bacillus subtilis* (Supplementary Figure S3), as witnessed by clear inhibition zones. These results confirm the formation of (Me)Lan rings with correct configurations in the arginine-containing lacticin 481 analogues.

### Combinatorial Biosynthesis of Ornithine-Containing Lacticin 481 Analogues

Previously, a hybrid leader for recognition by SyncM and the peptide epimerase OspD has been designed, based on the system of the wild-type precursor protein OspA.^41^ In this study, we further investigated whether this hybrid leader sequence could also be recognized by the peptide arginase OspR to modify the arginine-containing lacticin 481 analogues. For the combinatorial biosynthesis of ornithine-containing lacticin 481 analogues using OspR and SyncM, we first investigated the modification order, considering the substrate specificity of the enzymes. Since it was previously reported that OspR prefers to catalyze cyclic peptide substrates,^43^ we first attempted modification of the lacticin 481 precursor peptides by SyncM, followed by modification by OspR (Figure 3A, route 1). To be able to control the order of expression of SyncM and OspR, the *ospR* gene was cloned into the pBAD vector, under an arabinose inducible promoter, yielding the pBAD-OspR construct. The *syncM* gene was cloned into the pRSFDuet vector, producing pRSFDuet-SyncM. The precursor peptide (analogues) were constructed in a pCDFDuet vector. For the latter two constructs, IPTG was used to induce expression. These three constructs were cotransformed (Figure 3B 1), and expression of the precursor peptide and SyncM was induced first with IPTG, followed by expression of OspR with arabinose. Analysis by MALDI-TOF mass spectrometry (Supplementary Figure S4 and Table S3) indicates modification by SyncM, but the incorporation of ornithine was not observed.

In view of the above-mentioned absence of ornithine incorporation for the first approach, first expression SyncM and thereafter OspR, the order was inversed. In this second approach the precursor peptide and OspR were expressed, followed by expressing SyncM (Figure 3A 2). For this route, OspR was cloned into MCS2 of pCDFDuet vectors already containing the precursor peptide (analogues) in MCS1, yielding pCDFDuet-lacticin 481-x-OspR vectors, where x is either wild-type, H8R, H12R or H8&12R. These constructs were cotransformed with pBAD-SyncM. This time, analysis by MALDI-TOF mass spectrometry demonstrated both modification by SyncM as well as successful ornithine incorporation, indicating successful production of ornithine-containing lacticin 481 analogues (Figure 4 and Table S4). The cyclization states of the peptides were determined by reaction of the free cysteine thiols in the (with LahT150) digested precursor peptides with iodoacetamide (IAA). (Figure S5 and Table S5). As we predicted, SyncM exhibits the capability to catalyze ring formation even when noncanonical amino acids are present near the selected sites. This result is indicative of opportunities for incorporating ornithine into other positions of lacticin 481. Additionally, antimicrobial activity assays also demonstrated the correct stereochemical configuration of the lanthionine and methyllanthionine rings (Figure 5). This is the first time that the noncanonical amino acid ornithine is introduced in lantibiotics *in vivo*, especially directly flanking or within the methyllanthionine and lanthionine ring positions. Notably, OspR converted arginine residues at both position 8 (Figure 4B) and position 12 (Figure 4C) to ornithines. These data also demonstrate the ability to simultaneously incorporate ornithines in both positions 8 and 12 (Figure 4D).

Overall, these results demonstrate SyncM’s ability to modify ornithine-containing lacticin 481 analogues, highlighting its broad substrate tolerance.^19^ In contrast, OspR fails to modify positions 8 and 12 in cyclized precursor lacticin 481 products. Our findings suggest a different preferred order of post-translational modifications compared to the model proposed by Mordhorst et al.^43^ Their findings implied a stepwise progression of post-translational modifications in the OspA precursor protein, beginning with the OspD epimerase, followed by the OspM lanthionine synthetase, and concluding with the OspR arginase, proceeding from the N-terminus to the C-terminus. In this way, ornithine residues were installed flanking lanthionines. Our findings suggest that OspR prefers linear substrates over cyclized ones and that its ability to convert arginine residues into ornithines depends on the peptide structure.

### Antimicrobial Activity Evaluation of the Ornithine-Containing Lacticin 481 Analogues

For the antimicrobial activity assay, core peptides were purified by HPLC and the fractions containing the target masses were combined and freeze-dried (Figure S6–S9). All analogues exhibit antimicrobial activity against *B. subtilis* (Figure 5). The minimal inhibitory concentrations (MIC) of the ornithine-containing analogues compared to the wild-type clearly demonstrated that activity was maintained for H8O and only slightly reduced for H12O and the combined H8O & H12O (Table 1). Hence, the overall activity remained largely unchanged. The antibacterial activity of lacticin 481 is primarily driven by three interconnected thioether rings. However, we anticipate that replacing the hydrophobic amino acid chain labeled as R with Orn could modulate antibacterial effectiveness by modulating interactions with bacterial cell membranes. This potential improvement warrants further investigation through lacticin 481 biosynthesis studies. Moreover, we anticipate that this method and the used coexpression system can also be applied for the insertion of ornithine at other positions within lacticin 481 or other lanthipeptides, since a possible steric hindrance by ring formation would not play a role. Overall, our findings demonstrate the feasibility of combining post-translational modifications by OspR and SyncM to generate novel lacticin 481 analogues.

**Table 1:** Estimated Minimal Inhibitory Concentration (MIC) Values of Wild-Type Lacticin 481 and the Ornithine-Containing Lacticin 481, H8O, and H12O Analogues against *Bacillus subtilis* 168

**Analogue**	**MIC (**μg mL^–1^)
**Wild-type**	**4.17**
**H8O**	**4.17**
**H12O**	**8.33**
**H8&12O**	**8.33**

## Conclusions

This study demonstrates the successful production of active ornithine-containing lacticin 481 analogues by heterologous expression of SyncM and OspR in *E. coli*. These results provide a basis for the generation of lantibiotic derivatives containing ornithine residues, as an addition to the tools for lantibiotic peptide engineering. Moreover, our findings shed light on the substrate specificity of SyncM and OspR, underscoring SyncM’s broad substrate tolerance for modifying ornithine-containing peptides while suggesting OspR’s preference for linear lantibiotic substrates. Optimization of the hybrid leader peptide could possibly increase the modification efficiency of OspR and further analyses could confirm the effects of ornithine residues on the antimicrobial activity and other biological and physicochemical properties. This study not only contributes to a fundamental understanding of the biosynthesis of ornithine-containing lacticin 481 derivates, but also highlights the potential of this expression system for the expansion of the chemical diversity of lantibiotics. Such efforts may lead to the discovery of derivatives with potentially improved pharmacokinetic properties, enhancing the suitability for therapeutic applications.

## Materials & Methods

### General Experimental Procedures

Chemicals were purchased from Merck unless specified otherwise. Oligonucleotide primers were acquired from Biolegio (Nijmegen, The Netherlands). Bacto Tryptone, Bacto yeast extract and glycerol were purchased from BOOM B.V. Antibiotics were used at a final concentration of 50 μg/mL for spectinomycin (Merck) and kanamycin (Formedium), and 100 μg/mL for ampicillin (Formedium). All plasmids were cloned in *E. coli* Top10. For expression studies, *E. coli* BL21DE3 was used. Strains were grown in LB broth (Formedium, Norfolk, United Kingdom) at 37 °C, at 220 rpm, or on LB agar (Formedium, Norfolk, United Kingdom), unless specified otherwise. IPTG and arabinose were obtained from ThermoFisher.

### Molecular Cloning

The gene encoding the lacticin 481 core sequence was cloned into the pCDFDuet-leader vector, obtained from Molecular Genetics lab stock. The pCDFDuet-lacticin481 plasmid was used for generation of the desired analogues by site-directed mutagenesis with overlapping primer pairs,^49^ yielding pCDFDuet-lacticin481-analogue plasmids. The pRSFDuet-SyncM, pBAD-SyncM and pBAD-OspR plasmids were obtained from Molecular Genetics lab stock. The SyncM gene was previously amplified from a plasmid constructed by Arias-Orozco and co-workers in previous work.^23^ The OspR gene obtained from Molecular Genetics lab stock, which was constructed by Fleur based on a constructed plasmid by Bösch and co-workers.^41^ The *ospR* gene was cloned into the pCDF-lacticin 481-analogue plasmids, yielding pCDFDuet-lacticin481-OspR analogue plasmids. All plasmid constructs were confirmed by DNA sequencing (Macrogen Europe, Amsterdam, The Netherlands). Primers used in this study are summarized in Supplementary Table S1.

### Expression of Precursor Peptides with OspR and SyncM

The constructed pCDFDuet-lacticin 481 analogue-OspR plasmids were transformed into chemically competent *E. coli* BL21(DE3) cells containing the pBAD-SyncM plasmid, allowing expression of the precursor peptide analogue and OspR first (IPTG inducible promoter), followed by SyncM (arabinose inducible promoter). The pCDFDuet-lacticin 481 analogue plasmids were cotransformed with pBAD-OspR into chemically competent *E. coli* (BL21DE3) cells containing the pRSF-SyncM plasmid, allowing expression of the precursor peptide analogue and SyncM (IPTG inducible promoter) first, followed by OspR (arabinose inducible promoter). Transformed cells were plated on LB plates with the appropriate antibiotic. Single colonies were inoculated in 4 mL LB Broth, supplemented with the appropriate antibiotics at 37 °C, 220 rpm, overnight. The overnight culture was diluted 1:50 in Terrific Broth (TB: 24 g/L Bacto Yeast extract, 12 g/L Bacto Tryptone, 5 mL/L glycerol, 0.017 M KH_2_PO4, 0.072 M K_2_HPO4), supplemented with 1:1000 appropriate antibiotic. Cells were grown at 37 °C, 220 rpm to an OD_600_ of 1.0–2.0. The cultures were cooled on ice, followed by protein and peptide expression by induction with 1 mM IPTG (final concentration), and subsequent induction with 0.2 w/v% arabinose. Expression of OspR (with or without precursor peptide) was performed at 16 °C for ∼24 h, 200 rpm, and expression of SyncM (with or without precursor peptide) at 18 °C for ∼20 h, 200 rpm.

### Peptide Purification

The cells (from 100 mL culture) were harvested by centrifugation (4 °C, 8,500 rpm, 5 min), resuspended in 20 mL lysis buffer (20 mM NaH_2_PO4, 300 mM NaCl, 10 mM imidazole, pH 7.4), and lysed by sonication (10 s ON, 10 s OFF, 45–55% amplitude, 10–15 min). The lysate was obtained by centrifugation (4 °C, 10,000 rpm, 30 min) and filtered through 0.45 μM filters. The sample was loaded on an equilibrated Ni-NTA agarose column and mixed well. The resin was washed with 10 CV wash buffer (20 mM NaH_2_PO4, 300 mM NaCl, 40 mM imidazole, pH 7.4) and eluted with 5 mL elution buffer (20 mM NaH_2_PO4, 300 mM NaCl, 500 mM imidazole, pH 7.4). Peptide yields and purity were estimated by Tricine SDS-PAGE. The sample was desalted through an equilibrated PD-10 desalting column with Sephadex G-25 resin (GE Healthcare) and eluted in 7 mL 50 mM Tris-HCl pH 8.0. Core peptide was released from the His_6_-tagged leader by 1:20 addition of LahT150 protease (containing 1 mM DTT) for 2 h at 37 °C. The LahT150 protease was purified from *E. coli* containing the pETDuet-LahT150 construct according to the protocol described previously by Bobeica and co-workers.^50^ Core peptide mixtures were centrifuged (4 °C, 10,000 rpm, 15 min), filtered through 0.45 μM filters, and further purified with a second equilibrated Ni-NTA agarose column. The sample was loaded, mixed well and the flow-through was collected directly, followed by addition of 7 mL second His_6_-tag buffer (20 mM Tris, 300 mM NaCl, pH 7.5).

Core peptides were further purified by an open column with C18 resin (Waters), washed with 3 mL 0.1% trifluoroacetic acid (TFA) in acetonitrile (ACN) and equilibrated with 5 mL Milli-Q + 0.1% TFA. After sample loading, the column was washed with 10 mL 10% ACN + 0.1% TFA, and core peptide was eluted with 10 mL 60% ACN + 0.1% TFA and lyophilized. Lacticin 481 analogues produced by coexpression of OspR, followed by SyncM, were further purified by an Agilent Infinity HPLC system with a reverse-phase C18 column (Phenomenex Aeris 250 × 4.6 mm^2^, 3.6 μm particle size, 100 Å pore size). The system was first washed with 95% solvent B (ACN (HPLC-grade, VWR International) + 0.1% TFA) and 5% solvent A (Milli-Q + 0.1% TFA). Peptide fractions were collected through a linear gradient from 20 to 60% solvent B, and analyzed by MALDI-TOF mass spectrometry. The fractions containing the peptides with target masses were combined and freeze-dried.

### MALDI-TOF Mass Spectrometry

For MALDI-TOF analysis, 1 μL sample (after C18 column purification or HPLC) was spotted and dried on the MALDI target plate. Subsequently, 1 μL matrix solution (5 mg/mL α-cyano-4-hydroxycinnamic acid (Sigma-Aldrich) in 50% ACN + 0.1% TFA) was spotted on top of the sample. Matrix-assisted laser desorption ionization-time-of-flight (MALDI-TOF) mass spectrometry analysis was performed using a 4800 Plus MALDI TOF/TOF Analyzer (Applied Biosystems) in the reflector positive mode.

### Iodoacetamide (IAA) Assay

The cyclization states of the peptides were determined by reaction of the free cysteine thiols in the (with LahT150) digested precursor peptides with iodoacetamide (IAA). Addition of IAA only occurs when the cysteine thiol is available for reaction, indicating that the respective (methyl)lanthionine ring is not formed, yielding partially cyclized peptide products. For the IAA reaction, 10 μL of filtered digested precursor peptide together with 27.5 μL Milli-Q was treated with 2.5 μL of 20 mM freshly prepared TCEP (dissolved in Milli-Q, added to a final concentration of 1 mM TCEP), and allowed to react for 3h at room temperature. Subsequently, 10 μL of 50 mM freshly prepared IAA (dissolved in 50 mM Tris-HCl, pH 8.0, to a final concentration of 10 mM IAA) was added to the reaction mixture (total volume 50 μL), followed by incubation in the dark for 1h at room temperature. For MALDI-TOF mass spectrometry analysis, 1 μL of the reaction mixture was applied to the MALDI-TOF target plate. Each addition of IAA to a free cysteine thiol increases the monoisotopic mass of a peptide by 57.07 Da.

### Antimicrobial Activity Assay and MIC Test

The antimicrobial activity test agar plate was prepared by diluting overnight grown *Bacillus subtilis 168* culture in a mixture of 70% LB-agar and 30% LB, which was poured on a plate and dried at the flame. The freeze-dried products of the lacticin 481 analogues were dissolved in Milli-Q, spotted on the plate and allowed to dry. Plates were incubated at 37 °C for around 6 h to overnight. The Minimal Inhibitory Concentration (MIC) values of the lacticin 481 analogues were determined with *Bacillus subtilis 168*, and performed in triplicates using the 96-well plate broth microdilution method described by Wiegand et al.^51^ The freeze-dried lacticin 481 analogue products from HPLC were dissolved in Milli-Q to a final concentration of 50 μg/mL and diluted in Mueller Hinton Broth via serial dilution. The starting concentration of the overnight grown *Bacillus subtilis* culture was adjusted to approximately 5 × 10^5^ CFUs per mL. After approximately 18 h of incubation at 37 °C, the peptide concentration in the first well that shows no visible growth of the strain was defined as the MIC value.

## Supporting Information Available

Overview of the primers used, HPLC spectrum of lacticin 481 and mutants, additional MALDI-TOF mass spectrometry data, antimicrobial activity assays (PDF)
