# Supplementary material for: Biosynthesis of Antimicrobial Ornithine-Containing Lacticin 481 Analogues by Use of a Combinatorial Biosynthetic Pathway in Escherichia coli
Source: ACS Synth Biol. 2024 Dec 11;13(12):4209–17. doi: 10.1021/acssynbio.4c00650 (PMC11669161; doi:10.1021/acssynbio.4c00650)
Supplement: Supplementary file 1 — sb4c00650_si_001.pdf [file sb4c00650_si_001.pdf]

Supporting Information for Publication

Biosynthesis of antimicrobial ornithine-containing lacticin 481 analogues by use of a combinatorial biosynthetic pathway in *Escherichia coli*

Yanli Xu[1], Roos Reuvekamp[1], Oscar P. Kuipers[1,*]

[1]Department of Molecular Genetics, Groningen Biomolecular Sciences and Biotechnology Institute, University of Groningen, Groningen, 9747 AG, The Netherlands.

*Correspondence: o.p.kuipers@rug.nl (Oscar P. Kuipers)

Table S1. Primers used in this study.

Primer	Sequence (5' to 3')	Purpose
H8R-lac-Fw	CTGGTGTTATCCGTACCATCTCTCACGAATGCAACATGAACTCTTG	pCDF-lacticin481-H8R construct
H8R-lac-Rv	ATGGTACGGATAACACCAGAACCACCTTTGCCACCGCTGGCT	pCDF-lacticin481-H8R construct and pCDF-lacticin481-H8&12R construct
H12R-lac-Fw	TCTCGTGAATGCAACATGAACTCTTGGCAGTTCGTTTTC	pCDF-lacticin481-H12R construct
H12R-lac-Rv	ATGTTGCATTCACGAGAGATGGTGTGGATAACACCAGAA	pCDF-lacticin481-H12R construct
H8&12R-lac-Fw	CTGGTGTTATCCGTACCATCTCTCGTGAATGCAACATGAACTCTTG	pCDF-lacticin481-H8&12R construct
OspR-Fw	ATGGCTAAGATTCCGTTTTATATCATGG	PCR amplification of the OspR gene
OspR-Rv	TTAGACCTCTACTAAAACTTTTTTAGACAG	PCR amplification of the OspR gene
pCDF-T72-OspR-Fw	AAGTTTTAGTAGAGGTCTAAGCAGATCTCAATTGGATATCGGCCG	PCR amplification of the vectors (H8R, H12R and H8&12R analogues)
pCDF-T72-OspR-Rv	TAAAACGGAATCTTAGCCATATGTATATCTCCTTCTTATACTTAACTAATATACTAAGATGGGG	PCR amplification of the vectors (H8R, H12R and H8&12R analogues)

Table S2. Overview of the expected masses and the detected masses for the lacticin 481 arginine contained analogues produced with expression of SyncM (**Figure S2**). ND, not detected in the respective sample.

	Wild-type Expected	Wild-type Detected	H8R/H12R Expected	H8R Detected	H12R Detected	H8&12R Expected	H8&12R Detected
**Original mass**	2973.36	ND[a]	2992.41	ND	ND	3011.46	ND
**-1 H_2O**	2955.36	ND	2974.41	ND	ND	2993.46	ND
**-2 H_2O**	2937.36	2937.39	2956.41	ND	2954.29	2975.46	2972.28
**-3 H_2O**	2919.36	2918.41	2938.41	2938.35	2937.30	2957.46	2956.28
**-4 H_2O**	2901.36	2900.41	2920.41	2919.35	2919.30	2939.46	2938.28
**-5 H_2O**	2883.36	2882.40	2902.41	2901.35	2901.29	2921.46	2920.28

Table S3. Overview of the expected masses and the detected masses for the lacticin 481 analogues produced by expression of SyncM first, followed by OspR (**Figure S4**). ND, not detected in the respective sample.

	Wild-type Expected	Wild-type Detected	H8R/H12R Expected	H8R Detected	H12R Detected	H8&12R Expected	H8&12R Detected
**Original mass**	2973.36	ND[a]	2992.41	ND	ND	3011.46	ND
**-1 H_2O**	2955.36	ND	2974.41	ND	ND	2993.46	ND
**-2 H_2O**	2937.36	2935.69	2956.41	2955.57	2956.54	2975.46	ND
**-3 H_2O**	2919.36	2918.70	2938.41	2937.60	2937.61	2957.46	2956.88
**-4 H_2O**	2901.36	2900.70	2920.41	2919.65	2919.65	2939.46	2938.91
**-5 H_2O**	2883.36	2882.68	2902.41	2901.64	2901.66	2921.46	2920.89

Table S4. Overview of the expected masses and the detected masses for the lacticin 481 analogues produced by expression of OspR first, followed by SyncM (**Figure 4**). [a]ND, not detected in the respective sample.

	Wild-type Expected	Wild-type Detected	H8R/H12R Expected	H8R Detected	H12R Detected	H8&12R Expected	H8&12R Detected
**Original mass**	2973.36	2970.52	2992.41	ND[a]	ND	3011.46	ND
**-1 H_2O**	2955.36	2955.53	2974.41	2971.52	ND	2993.46	ND
**-2 H_2O**	2937.36	2935.51	2956.41	2955.56	ND	2975.46	ND
**-3 H_2O**	2919.36	2918.52	2938.41	2937.56	2937.66	2957.46	ND
**-4 H_2O**	2901.36	2900.52	2920.41	2919.56	ND	2939.46	ND
**-5 H_2O**	2883.36	2882.53	2902.41	2901.55	ND	2921.46	ND
**Original mass + 1 Orn**	-	-	2950.41	ND	ND	2969.46	ND
**-1 H_2O + 1 Orn**	-	-	2932.41	ND	ND	2951.46	ND
**-2 H_2O + 1 Orn**	-	-	2914.41	ND	2913.69	2933.46	2932.85
**-3 H_2O + 1 Orn**	-	-	2896.41	2896.55	2895.70	2915.46	2913.80
**-4 H_2O + 1 Orn**	-	-	2878.41	2877.55	2877.68	2897.46	2895.81
**-5 H_2O + 1 Orn**	-	-	2860.41	2859.56	2860.79	2879.46	2877.80
**Original mass + 2 Orn**	-	-	-	-	-	2927.46	ND
**-1 H_2O + 2 Orn**	-	-	-	-	-	2909.46	ND
**-2 H_2O + 2 Orn**	-	-	-	-	-	2891.46	2891.83
**-3 H_2O + 2 Orn**	-	-	-	-	-	2873.46	2872.81
**-4 H_2O + 2 Orn**	-	-	-	-	-	2855.46	2854.82
**-5 H_2O + 2 Orn**	-	-	-	-	-	2837.46	2838.85

Table S5. Overview of the expected masses and the detected masses for the lacticin 481 analogues produced by expression of OspR first, followed by SyncM, subjected to reaction with iodoacetamide (IAA). ND[a], not detected in the respective sample(Figure S10).

	Wild-type Expected	Wild-type Detected	H8R/H12R Expected	H8R Detected	H12R Detected	H8&12R Expected	H8&12R Detected
**Original mass**	2973.36	ND[a]	2992.41	ND	ND	3011.46	ND
**-1 H_2O**	2955.36	ND	2974.41	ND	ND	2993.46	ND
**-2 H_2O**	2937.36	2936.57	2956.41	2956.56	ND	2975.46	ND
**-3 H_2O**	2919.36	2918.51	2938.41	2937.55	ND	2957.46	ND
**-4 H_2O**	2901.36	2901.49	2920.41	2919.56	ND	2939.46	ND
**-5 H_2O**	2883.36	2883.50	2902.41	2901.53	ND	2921.46	ND
**-1 H_2O + IAA**	3012.43	ND	3031.48	ND	ND	3050.53	ND
**-2 H_2O + IAA**	2994.43	2993.56	3013.48	3012.59	ND	3032.53	ND
**-3 H_2O + IAA**	2976.43	2975.49	2995.48	2994.62	ND	3014.53	ND
**-4 H_2O + IAA**	2958.43	ND	2977.48	ND	ND	2996.53	ND
**-5 H_2O + IAA**	2940.43	ND	2959.48	ND	ND	2978.53	ND
**Original mass + 1 Orn**	-	-	2950.41	ND	ND	2969.46	ND
**-1 H_2O + 1 Orn**	-	-	2932.41	ND	ND	2951.46	ND
**-2 H_2O + 1 Orn**	-	-	2914.41	ND	2915.55	2933.46	2933.66
**-3 H_2O + 1 Orn**	-	-	2896.41	2896.51	2895.57	2915.46	2914.69
**-4 H_2O + 1 Orn**	-	-	2878.41	2877.55	2877.57	2897.46	2896.68
**-5 H_2O + 1 Orn**	-	-	2860.41	2859.55	ND	2879.46	ND
**-1 H_2O + 1 Orn + IAA**	-	-	2989.48	ND	ND	3008.53	ND
**-2 H_2O + 1 Orn + IAA**	-	-	2971.48	ND	2970.60	2990.53	2989.71
**-3 H_2O + 1 Orn + IAA**	-	-	2953.48	ND	2952.70	2972.53	2971.70
**-4 H_2O + 1 Orn + IAA**	-	-	2935.48	ND	ND	2954.53	ND
**-5 H_2O + 1 Orn + IAA**	-	-	2917.48	ND	ND	2936.53	ND
**Original mass + 2 Orn**	-	-	-	-	-	2927.46	ND
**-1 H_2O + 2 Orn**	-	-	-	-	-	2909.46	ND
**-2 H_2O + 2 Orn**	-	-	-	-	-	2891.46	ND
**-3 H_2O + 2 Orn**	-	-	-	-	-	2873.46	2872.67
**-4 H_2O + 2 Orn**	-	-	-	-	-	2855.46	2855.63
**-5 H_2O + 2 Orn**	-	-	-	-	-	2837.46	ND
**-1 H_2O + 2 Orn + IAA**	-	-	-	-	-	2966.53	ND
**-2 H_2O + 2 Orn + IAA**	-	-	-	-	-	2948.53	ND
**-3 H_2O + 2 Orn + IAA**	-	-	-	-	-	2930.53	ND
**-4 H_2O + 2 Orn + IAA**	-	-	-	-	-	2912.53	ND
**-5 H_2O + 2 Orn + IAA**	-	-	-	-	-	2894.53	ND

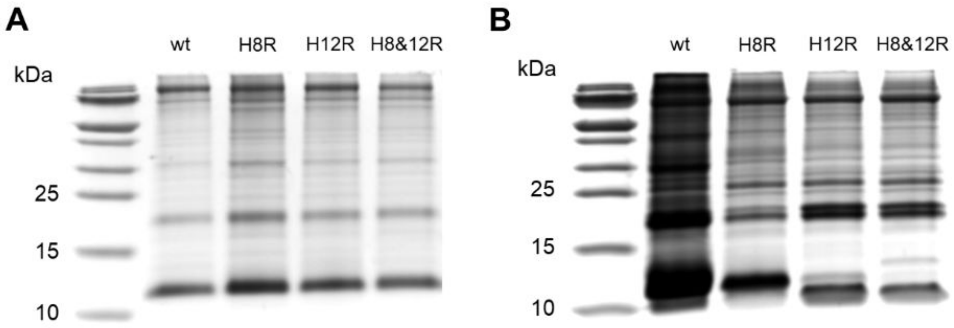

Figure S1. Expression of lacticin 481 analogues. Tricine SDS PAGE analysis of Ni-NTA His-tag elutions of wild-type (wt) lacticin 481 and the H8R, H12R and H8&12R analogues. **A**) Expression of precursor peptides with SyncM first, followed by OspR. **B**) Expression of precursor peptides with OspR first, followed by SyncM.

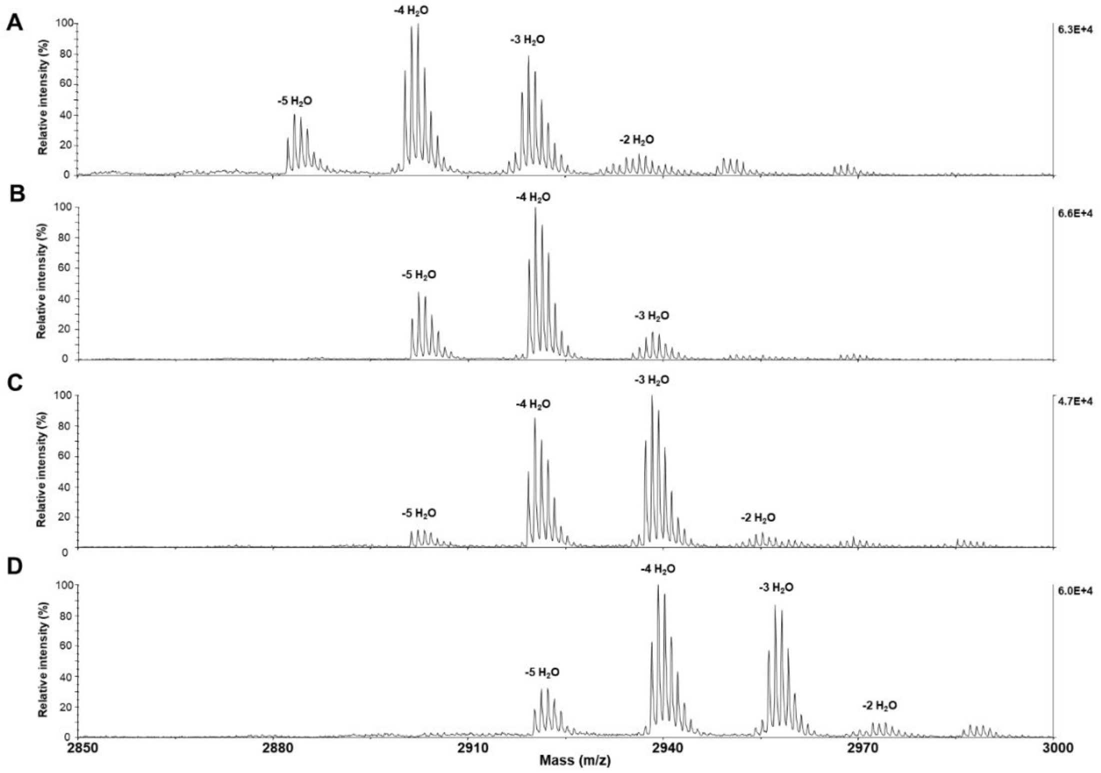

Figure S2. MALDI-TOF mass spectrometry analysis of arginine-containing lacticin 481 analogues modified by SyncM. Analysis of samples taken after C-18 desalting column purification. MALDI-TOF mass spectra of **A)** wild-type lacticin 481, **B)** the H8R lacticin 481 analogue, **C)** the H12R lacticin 481 analogue, and **D)** the H8&12R lacticin 481 analogue.

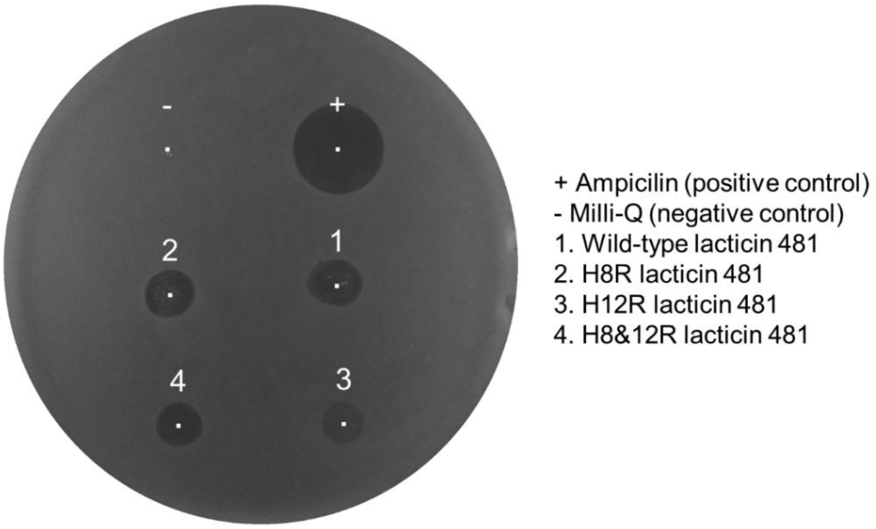

Figure S3. Antimicrobial activity of the arginine-containing lacticin 481 analogues against *B. subtilis 168*, including positive and negative controls.

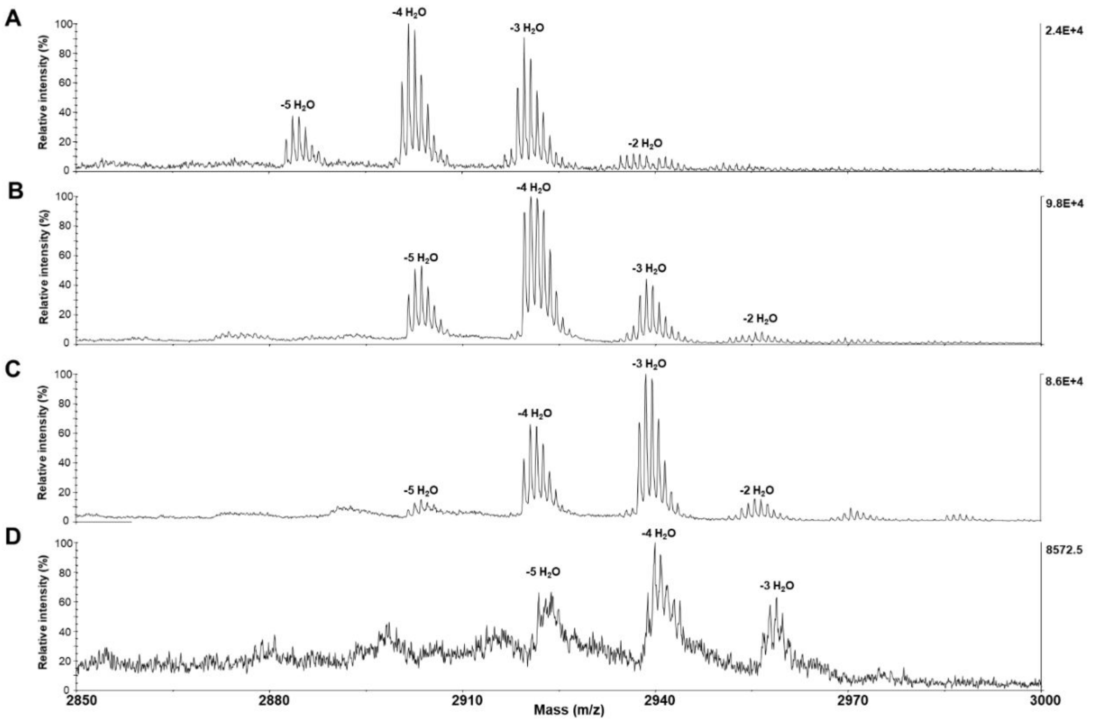

Figure S4. MALDI-TOF mass analysis of lacticin 481 analogues produced with expression of SyncM first, followed by OspR. Analysis of samples taken after C-18 desalting column purification. MALDI-TOF mass spectra of **A)** wild-type lacticin 481, **B)** the H8R lacticin 481 analogue, **C)** the H12R lacticin 481 analogue, and **D)** the H8&12R lacticin 481 analogue. For all lacticin 481 analogues, modification by OspR is not observed.

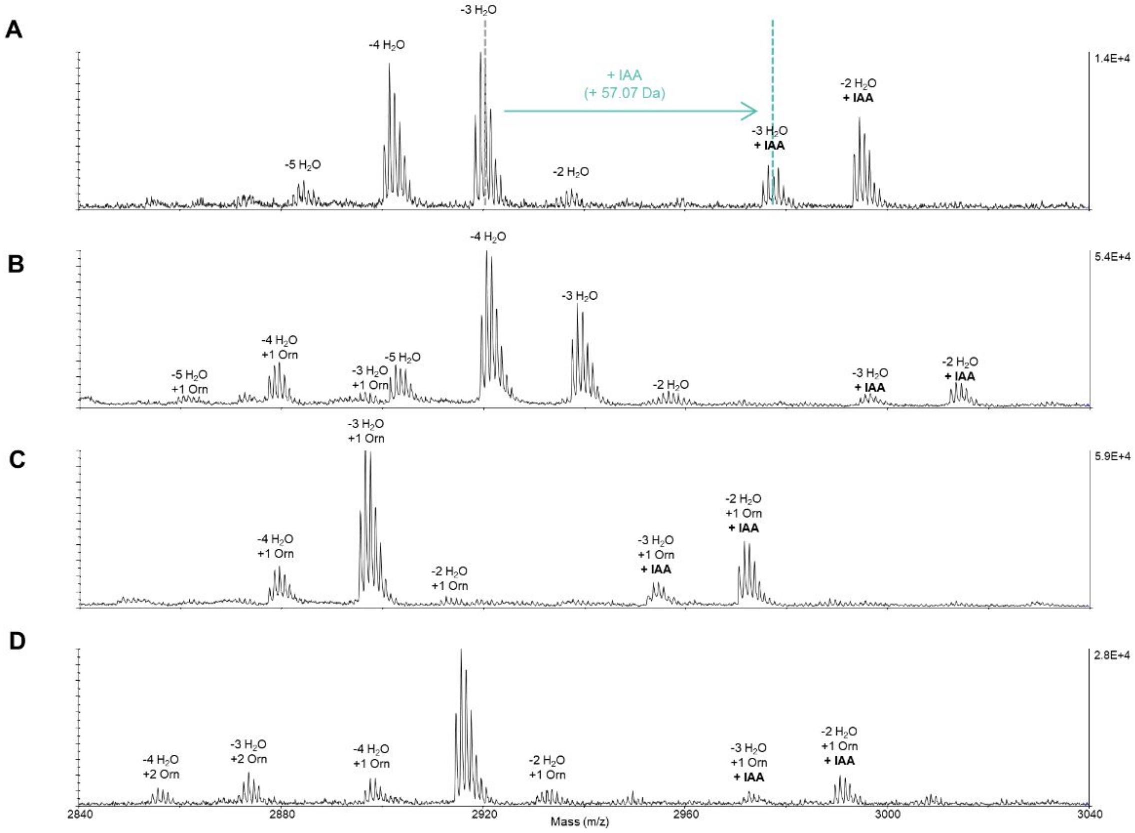

Figure S5. MALDI-TOF mass analysis of iodoacetamide alkylation reactions of lacticin 481 analogues produced by expression of OspR first, followed by SyncM, samples subjected to reaction with IAA. Iodoacetamide (IAA) reacts with free cysteine thiol residues, resulting in a mass addition of +57.07 Da. Peptide samples were taken after incubation with LahT150 and filtration through 0.45 μm filters. Modification of the lacticin 481 analogues by SyncM and OspR is observed, as well as the presence of partially cyclized peptide products, indicated by addition of IAA. MALDI-TOF mass spectra of A) wild-type lacticin 481, B) the H8R lacticin 481 analogue, C) the H12R lacticin 481 analogue, and D) the H8&12R lacticin 481 analogue.

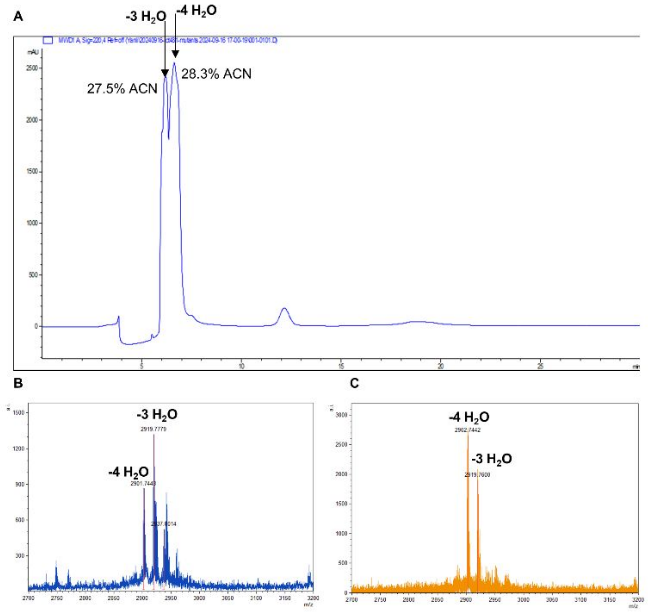

Figure S6. HPLC spectrum and corresponding MALDI-TOF mass analysis of wild type lct481. **A)** The HPLC spectrum of wild-type lct481was obtained at a detection wavelength of 220 nm with a flow rate of 1 mL/min. The fractions corresponding to the three- and four-dehydration modification states were collected and combined for subsequent experiments. **B)** MALDI-TOF mass analysis of corresponding to the mainly three and four dehydration state peaks identified in the HPLC analysis were obtained.

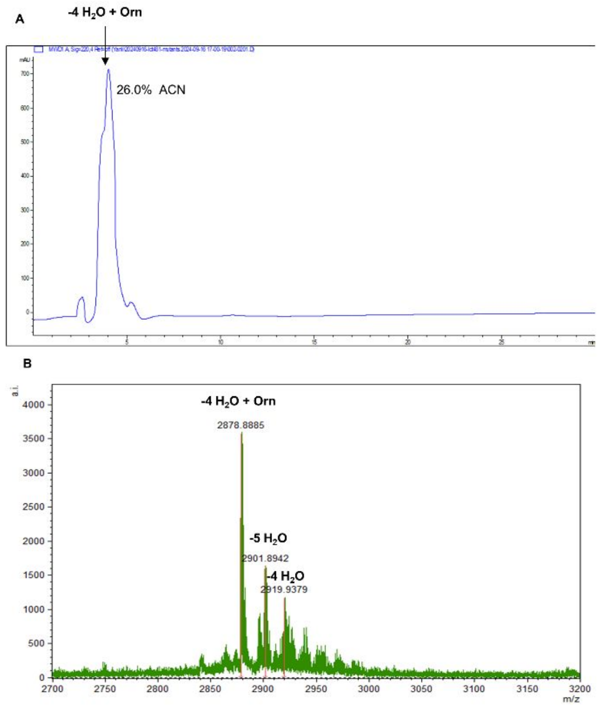

Figure S7. HPLC spectrum and corresponding MALDI-TOF mass analysis of lct481-8R. A) The HPLC spectrum of lct481-8R was obtained at a detection wavelength of 220 nm with a flow rate of 1 mL/min. The fractions corresponding to the four-dehydration modification state was collected and combined for subsequent experiments. B) MALDI-TOF mass analysis of corresponding to the mainly four dehydration state peaks with orn modifications identified in the HPLC analysis were obtained.

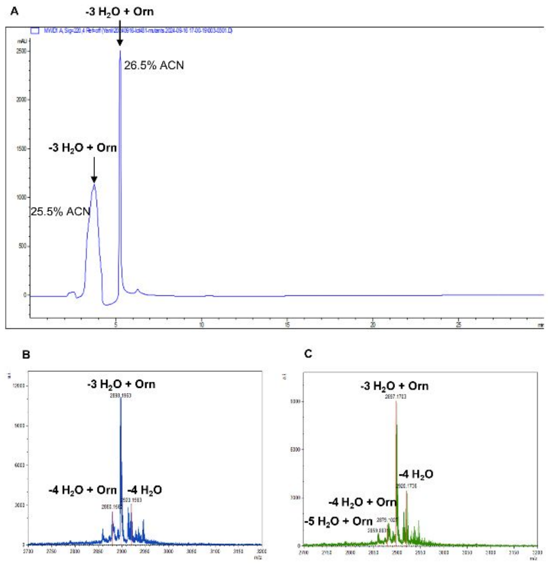

Figure S8. HPLC spectrum and corresponding MALDI-TOF mass analysis of lct481-12R. A) The HPLC spectrum of lct481-12R was obtained at a detection wavelength of 220 nm with a flow rate of 1 mL/min. The fractions corresponding to the three-dehydration modification state with orn modification were collected and combined for subsequent experiments. B) MALDI-TOF mass analysis of corresponding to the mainly three dehydration state peaks with orn modifications identified in the HPLC analysis were obtained.

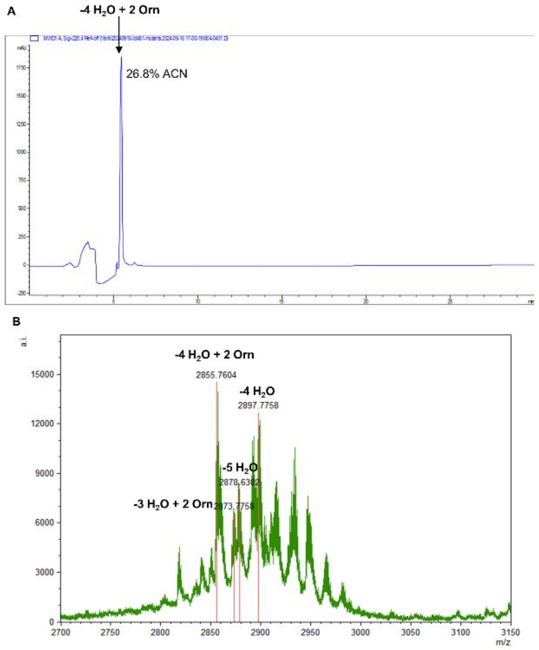

Figure S9. HPLC spectrum and corresponding MALDI-TOF mass analysis of lct481-8R-12R. A) The HPLC spectrum of lct481-8R-12R was obtained at a detection wavelength of 220 nm with a flow rate of 1 mL/min. The fractions corresponding to the four-dehydration modification state was collected and combined for subsequent experiments. B) MALDI-TOF mass analysis of corresponding to the mainly four dehydration state peaks with orn modifications identified in the HPLC analysis were obtained.

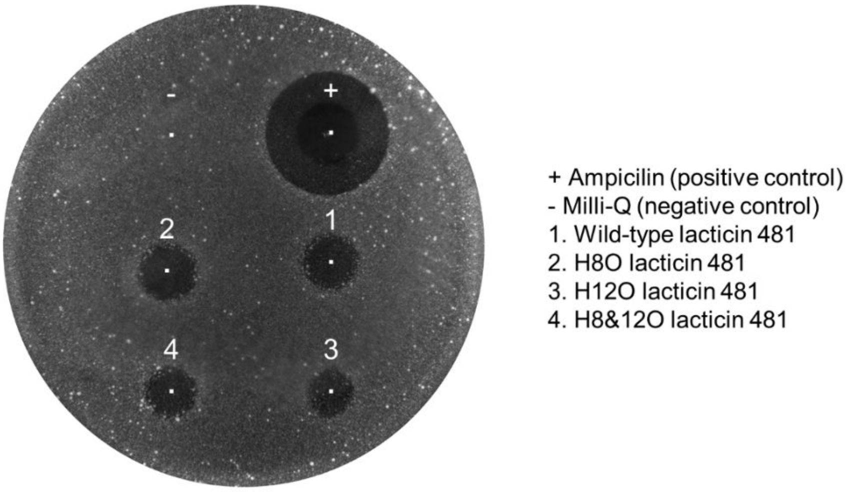

Figure S10. Antimicrobial activity of the ornithine-containing lacticin 481 analogues against *B. subtilis 168*, including positive and negative controls.
